# Supplementary material for: Multifaceted Defects in Monocytes in Different Phases of Chronic Hepatitis B Virus Infection: Lack of Restoration after Antiviral Therapy
Source: Microbiol Spectr. 2022 Nov 29;10(6):e01939-22. doi: 10.1128/spectrum.01939-22 (PMC9769680; doi:10.1128/spectrum.01939-22)
Supplement: Supplemental file 1 — Supplemental material. Download spectrum.01939-22-s0001.pdf, PDF file, 2.7 MB [file spectrum.01939-22-s0001.pdf]

Supplementary Information

Multifaceted defects in monocytes in different phases of chronic HBV infection: lack of restoration after antiviral therapy

Debangana Dey[1], Sourina Pal[1], Bidhan Chandra Chakraborty[2], Ayana Baidya[1], Soham Bhadra[1], Ranajoy Ghosh[3], Soma Banerjee[1], SK Mahiuddin Ahammed[4], Abhijit Chowdhury[4] and Simanti Datta[1]*

[1]Centre for Liver Research, School of Digestive and Liver Diseases, Institute of Post Graduate Medical Education and Research, Kolkata, India

[2]Multidisciplinary Research Unit, Institute of Post Graduate Medical Education and Research, Kolkata, India

[3]Division of Pathology, School of Digestive and Liver Diseases, Institute of Postgraduate Medical Education and Research, Kolkata, India

[4]Department of Hepatology, School of Digestive and Liver Diseases, Institute of Post Graduate Medical Education and Research, Kolkata, India

Medical Education and Research, Kolkata, India

Addresses correspondence to

Prof. Simanti Datta,

Centre for Liver Research, School of Digestive and Liver Diseases,

Institute of Post Graduate Medical Education and Research,

244, A.J. C. Bose Road, Kolkata-700020, India.

Tel- (91)-(033)-2223 5435; Fax- (91)-(033)-2223 6383

E-mail- seemdatt@gmail.com

Supplementary Table S1 Demographic, biochemical and virological characteristics of study subjects belonging to different phases in the natural history of chronic HBV infection and healthy controls

**Characteristics**	**HC** (N=19)	**IT** (N=10)	**EP-CHB** (N=15)	**IC** (N=22)	**EN-CHB** (N=18)
**Age, years Median (Range)**	30 (22-48)	12 (5-18)	28 (18-56)	38 (21-65)	36 (18-62)
**Sex (Male/Female)**	10:9	8:2	11:4	14:8	13:5
**HBeAg Status**	-	Positive	Positive	Negative	Negative
**ALT(IU/L) Median (Range)**	18 (14-22)	28 (17-36)	87 (49-147)	23 (12-32)	77 (43-137)
**HBV DNA (copies/ml) Median (Range)**	-	$8X10^{8}$ ($5X10^{7}$-$2.5X10^{9}$)	$6.4X10^{6}$ ($1.5X10^{5}$-$8X10^{7}$)	250 (250-$2.5X10^{3}$)	$4X10^{5}$ ($1.6X10^{4}$-$2X10^{7}$)

IT, Immunotolerant; EP-CHB, HBeAg-positive chronic hepatitis B; IC, Inactive carriers; ENCHB, HBeAg-negative chronic hepatitis B; HC, Healthy controls; ALT, alanine aminotransferase; IU, international unit;

Supplementary Table S2 List of reagents

SL No.	Reagent Name	Origin/Clone	Cat No.	Company name
1	Antihuman-CD14-FITC	M5E2	555397	BD Biosciences
2	Antihuman-HLA-DR-BV421	L243	307636	BioLegend Inc.
3	Antihuman-CD16-PECY7	3G8	557744	BD biosciences
4	Antihuman-TLR2-PE	TL2.1	309707	BioLegend Inc.
5	Antihuman-TLR4-APC	HTA125	312815	BioLegend Inc.
6	Antihuman-TLR8-PE	S16018A	395503	BioLegend Inc.
7	Antihuman-TLR9-APC	S16013D	394807	BioLegend Inc.
8	Antihuman-TNF-α-APC	MAb11	551384	BD Biosciences
9	Antihuman-IL-12-PE	C11.5	559329	BD Biosciences
10	Antihuman-IL-6-PE	MQ2-6A3	559331	BD Biosciences
11	Antihuman-LAP-TGF-β-PE	TW4-2F8	349604	BioLegend Inc.
12	Antihuman-IL-10-APC	JES3-19F1	506807	BioLegend Inc.
13	Antihuman-CD14-PerCP	HCD14	325632	BioLegend Inc.
14	Antihuman-CD64-PE	10.1	561926	BD Biosciences
15	FITC-zymosan particle	Zymosan prepared from yeast cell wall (*Saccharomyces cerevisiae*)	IAK0110	Sigma-Aldrich
16	2′,7′-Dichlorodihydrofluorescein diacetate (DCFH-DA)		D6883	Sigma-Aldrich
17	Antihuman-NOS2-AF647	C-11	SC-7271	Santa Cruz Biotechnology
18	Recombinant Hepatitis B Surface Antigen (Adw) protein	Expression system- *Pichia pastoris*	ab91276	abcam
19	β-galactosidase	*Aspergillus oryzae*	G5160	Sigma-Aldrich
20	Recombinant Human IL-4		766204	BioLegend Inc.
21	Antihuman-β-Catenin-PE	15B8	12-2567-41	Invitrogen
22	β-catenin/TCF inhibitor (iCRT3)		219332	Sigma-Aldrich
23	Recombinant M-CSF		574804	BioLegend Inc.
24	Lipopolysaccaride		L-4391	Sigma-Aldrich
25	Brefeldin A		B6542	Sigma-Aldrich
26	Recombinant IFN-γ		570204	BioLegend Inc.
27	Antihuman-CD68-FITC	Y1/82A	333806	BioLegend Inc.
28	Antihuman-CD4-FITC	RPA-T4	30056	BioLegend Inc.
29	Antihuman-CD25-PECY7	M-A251	561405	BD Biosciences
30	Antihuman-FoxP3-PE	259D/C7	560046	BD Biosciences
31	Antihuman-CXCR3-PE	1C6/CXCR3	560928	BD Biosciences
32	Antihuman-CXCR5-APC	J252D4	356907	BioLegend Inc.
33	Antihuman-CCR4-PE	L291H4	359411	BioLegend Inc.
34	Antihuman-CCR6-APC	G034E3	353415	BioLegend Inc.
35	Purified anti-CD3 antibody	HIT3a	300314	BioLegend Inc.
36	Purified anti-CD28 antibody	CD28.2	302914	BioLegend Inc.
37	Antihuman-CCR2-PE	K036C2	357206	BioLegend Inc.
38	Antihuman-CD33 PE	WM53	983904	BioLegend Inc.
39	LSM-1077		LS001	HiMedia

40	BD FACS Lysing solution		349202	BD Biosciences
41	BD sheath fluid		342003	BD Biosciences
42	BD cytofix/cytoperm		554722	BD Biosciences
43	BD cytofix/cytoperm wash		554723	BD Biosciences
44	True nuclear fix concentrate		73158	BioLegend Inc.
45	True nuclear permeabilization solution		73162	BioLegend Inc.
46	Roswell Park Memorial Institute (RPMI) 1640 Medium		11875-093	Gibco
47	Fetal Bovine Serum, heat inactivated		16140071	Gibco
48	Amphotericin B		15290-026	Gibco
49	Antibiotic/Antimycotic		15240-062	Gibco
50	CD14-Microbeads		130-050-201	Miltenyi Biotec
51	MS column		130-042-201	Miltenyi Biotec
52	TRIzol reagent		15596026	Invitrogen
53	RevertAid Reverse Transcriptase		EP0442	Thermo Scientific
54	Random Hexamer Primer		SO142	Thermo Scientific
55	RNase Inhibitor		BB X0010	BioBharati Lifescience Pvt. Ltd.
56	PowerUp SYBR Green Master mix		A25742	Applied Biosystems

Supplementary Fig. S1

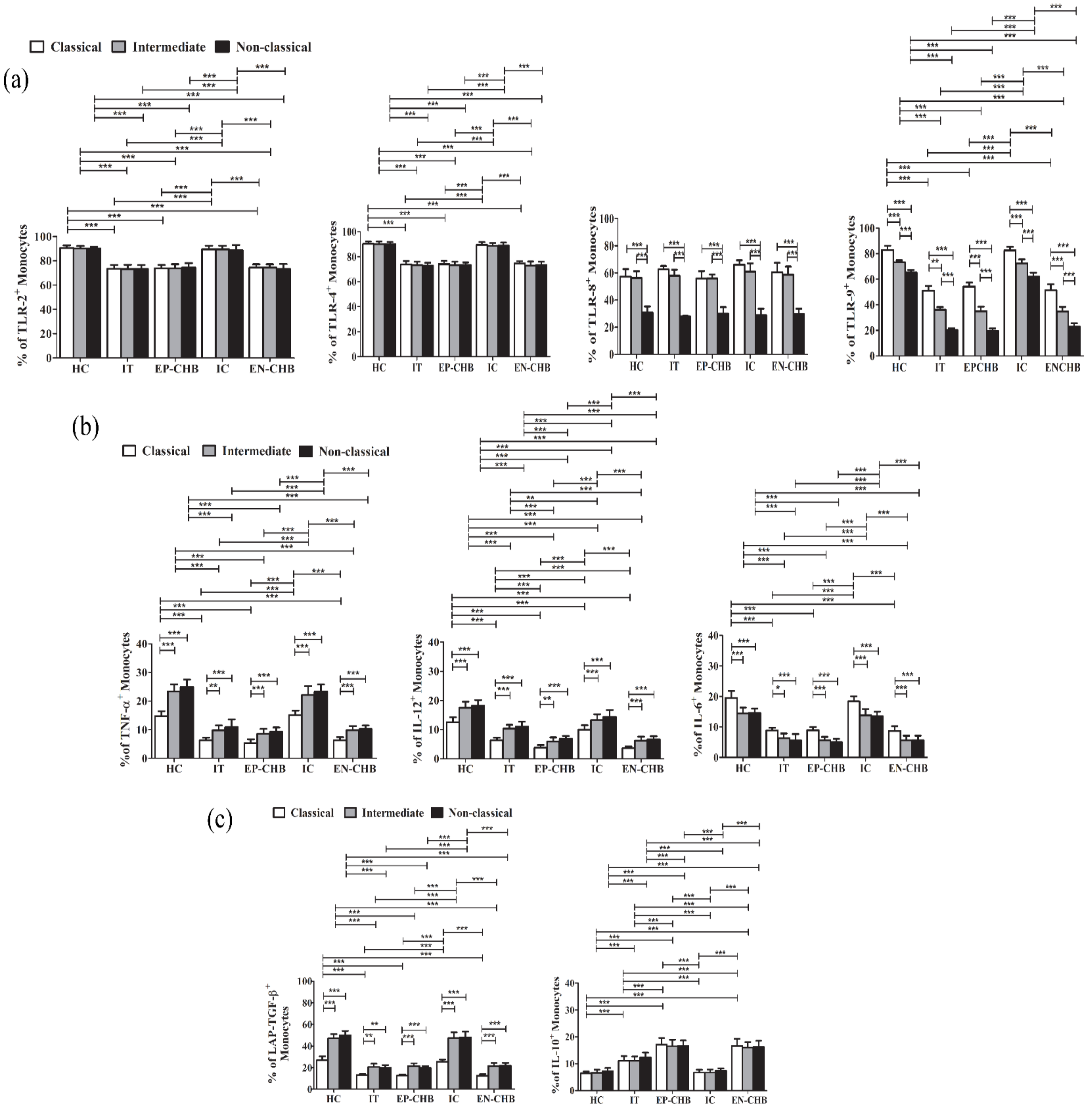

Fig. S1 Grouped bar diagrams demonstrating pooled data of percentages of classical, intermediate and non-classical monocytes expressing (a) TLR-2, TLR-4, TLR-8, TLR-9, (b) TNF-α, IL-12, IL-6 and (c) LAP-TGF-β, IL-10 in healthy controls (HC), Immune-tolerant (IT), HBeAg-positive chronic hepatitis B (EP-CHB), Inactive carriers (IC), and HBeAg-negative chronic hepatitis B (EN-CHB). Statistical significance was assessed by one way ANOVA test followed by Tukey's Multiple Comparison Test. (*$P < 0.05$, **$P < 0.001$ and ***$P < 0.0001$).

Supplementary Fig. S2

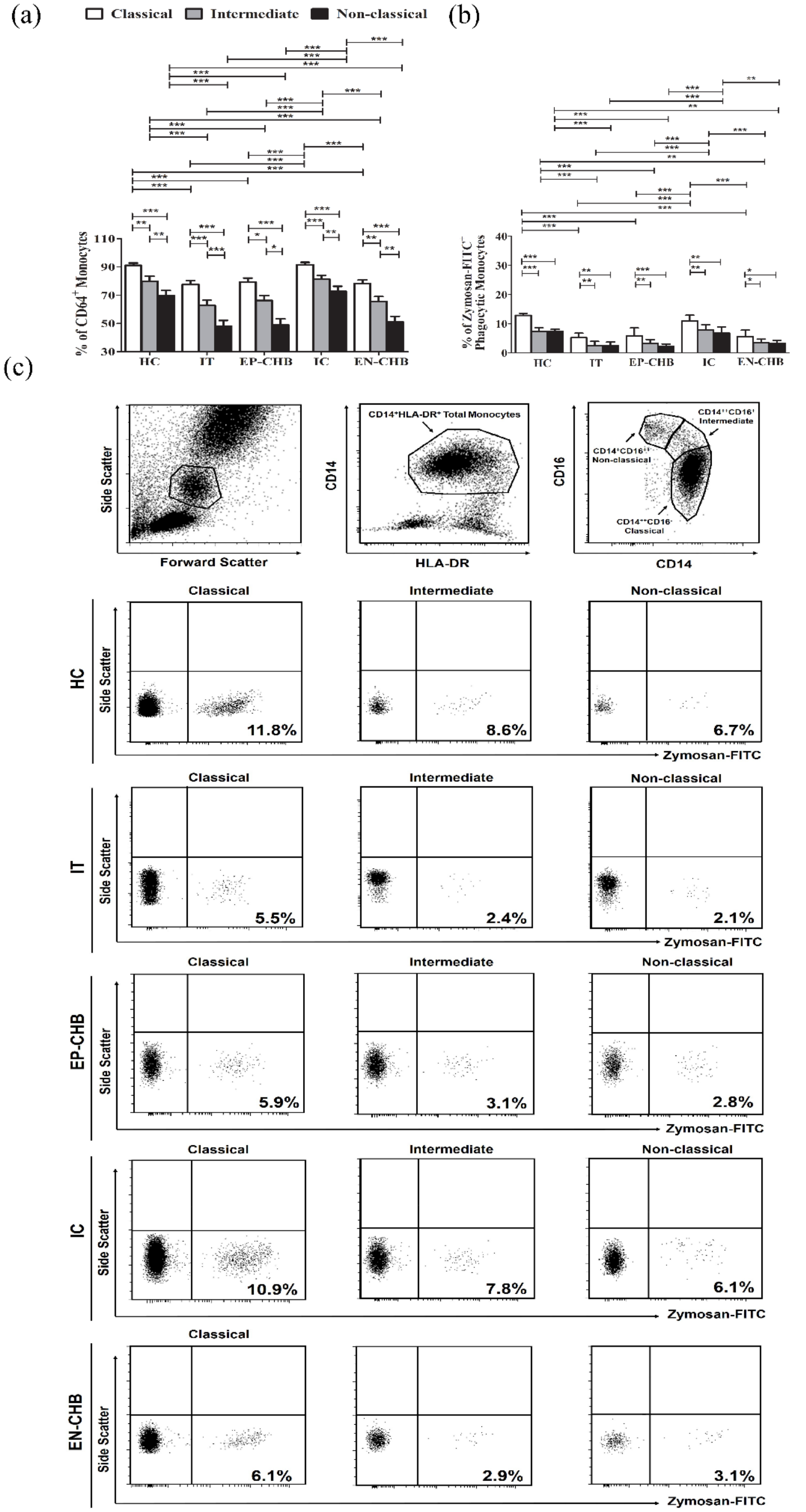

Fig. S2 Bar diagrams representing frequencies of classical, intermediate and non-classical monocytes (a) expressing CD64 and (b) with phagocytosed Zymosan-FITC particles in Immune-tolerant (IT), HBeAg-positive chronic hepatitis B (EP-CHB), Inactive carriers (IC), HBeAg-negative CHB (EN-CHB) and healthy controls (HC). (c) FACS plots showing percentages zymosan-FITC^{+} phagocytic monocyte-subsets (classical, intermediate and non-classical) in HC, IT, EP-CHB, IC and EN-CHB patient.

Supplementary Fig. S3

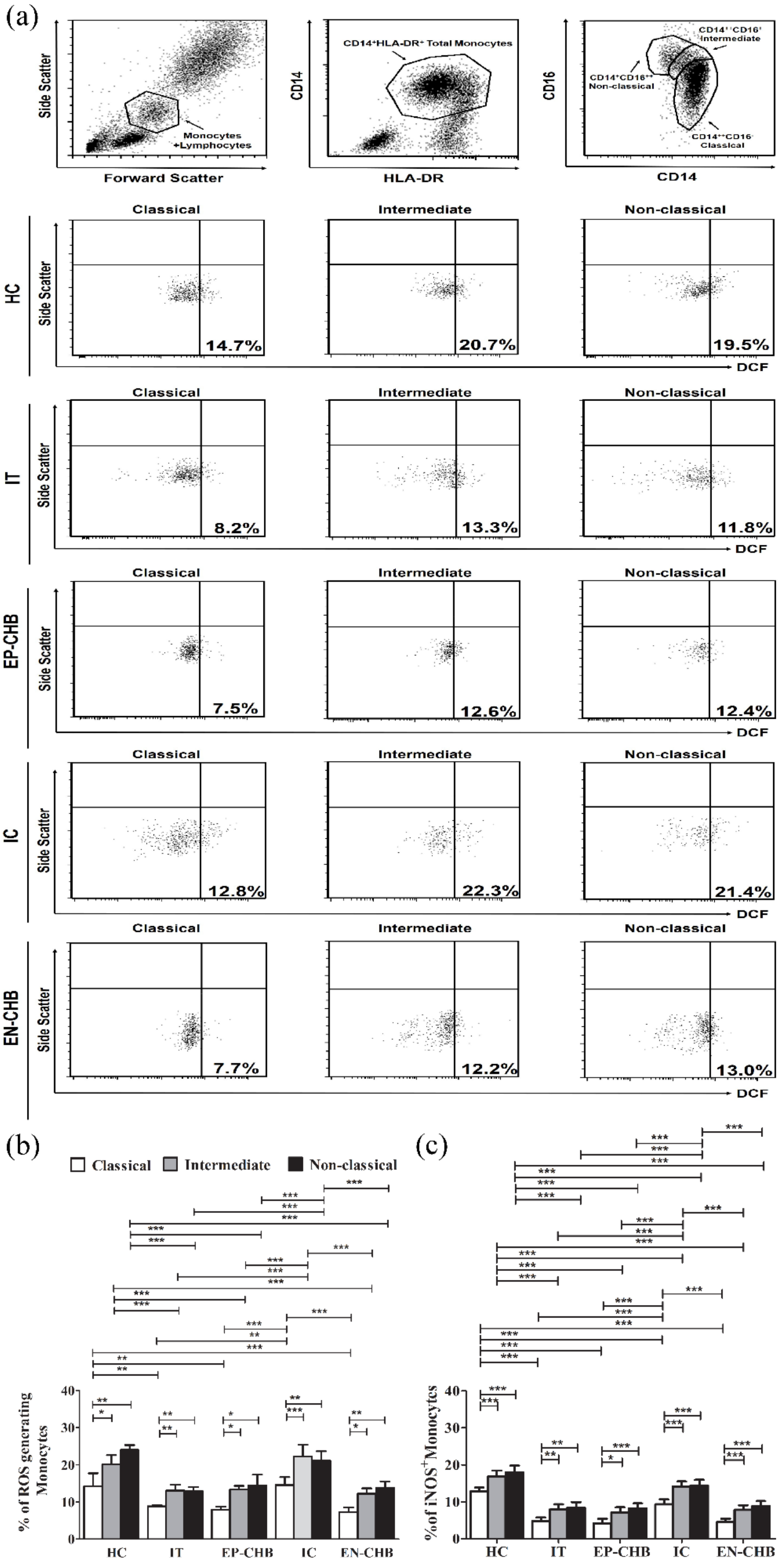

Fig. S3 (a) FACS plots showing percentages of classical, intermediate and non-classical monocytes generating reactive oxygen species (ROS) as measured by DCF fluorescence in healthy control (HC), Immune-tolerant (IT), HBeAg-positive chronic hepatitis B (EP-CHB), Inactive carrier (IC) and HBeAg-negative CHB (EN-CHB) patient. Bar diagrams representing frequencies of classical, intermediate and non-classical monocytes (b) generating ROS and (c) expressing iNOS in IT, EP-CHB, IC, EN-CHB and HC.

Supplementary Fig. S4

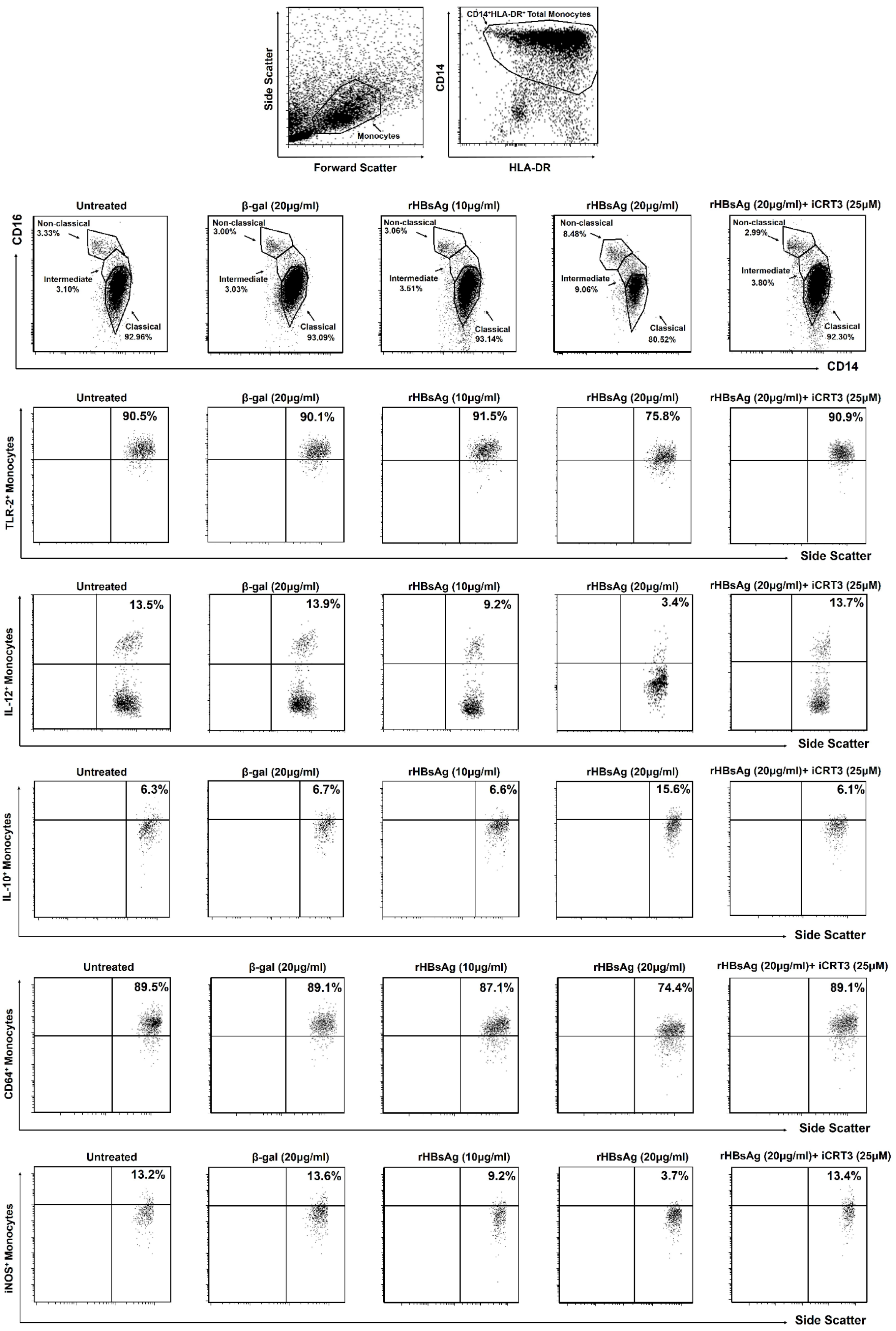

Fig. S4 Representative FACS plots displaying the distribution of monocyte subsets as well as the percentages of TLR-2/IL-12/IL-10/CD64/iNOS expressing monocytes following sorting of $CD14^+$ monocytes of healthy individual and treatment with β-galactosidase (β-gal) (20µg/ml), recombinant Hepatitis B surface antigen (rHBsAg) (10µg/ml and 20µg/ml), or combination of rHBsAg (20µg/ml) and β-catenin/TCF inhibitor (iCRT3) (25µM) for 48 hours.

Supplementary Fig. S5

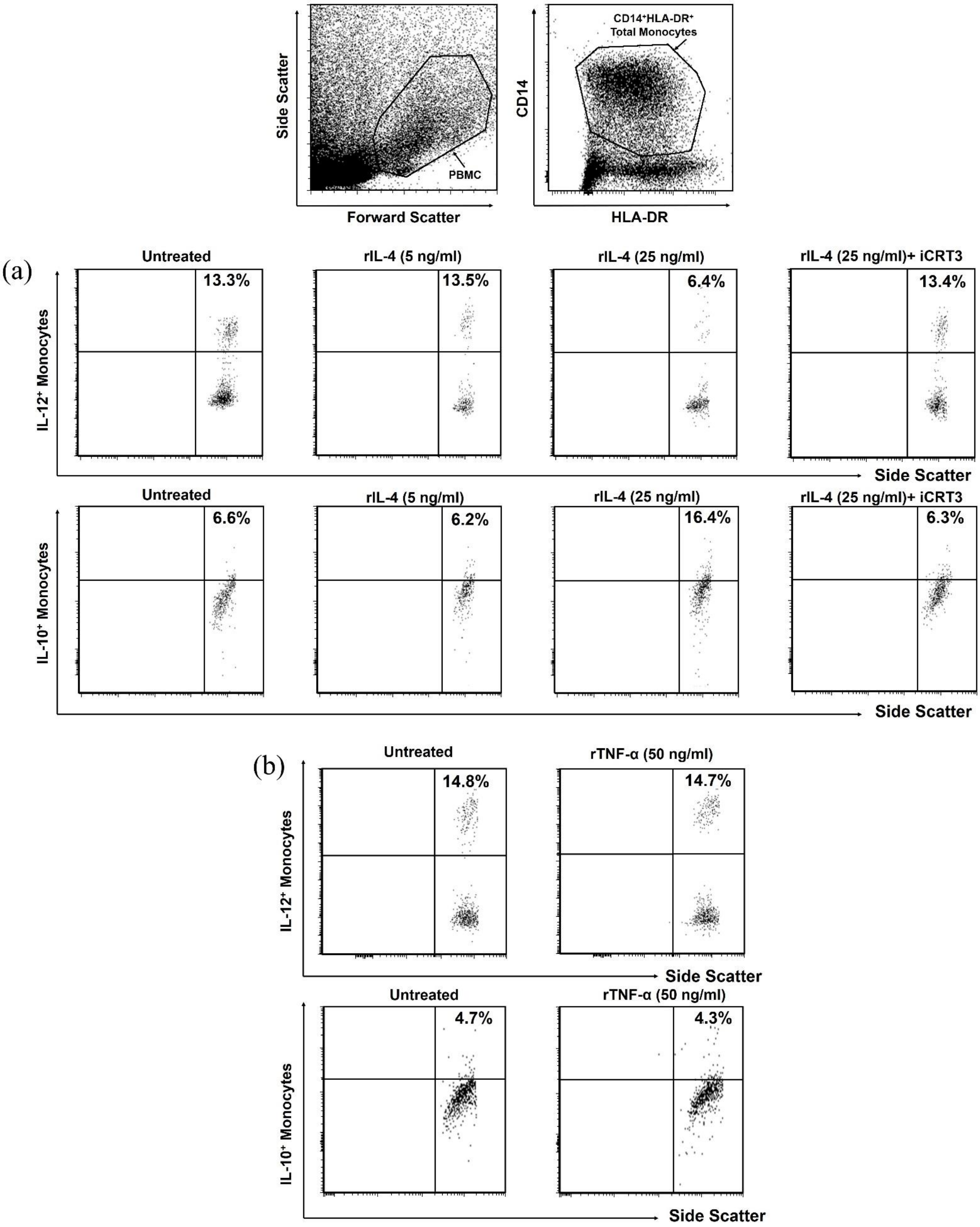

Fig. S5 Representative FACS plots displaying the frequencies of monocytes of healthy controls (HC) expressing IL-12 and IL-10 following treatment of PBMC with (a) recombinant IL-4 (rIL-4) (5ng/ml and 25μg/ml), or combination of rIL-4 (25ng/ml) and β-catenin/TCF inhibitor (iCRT3) (25μM) and (b) recombinant TNF-α (rTNF-α) (50ng/ml).

Supplementary Fig. S6

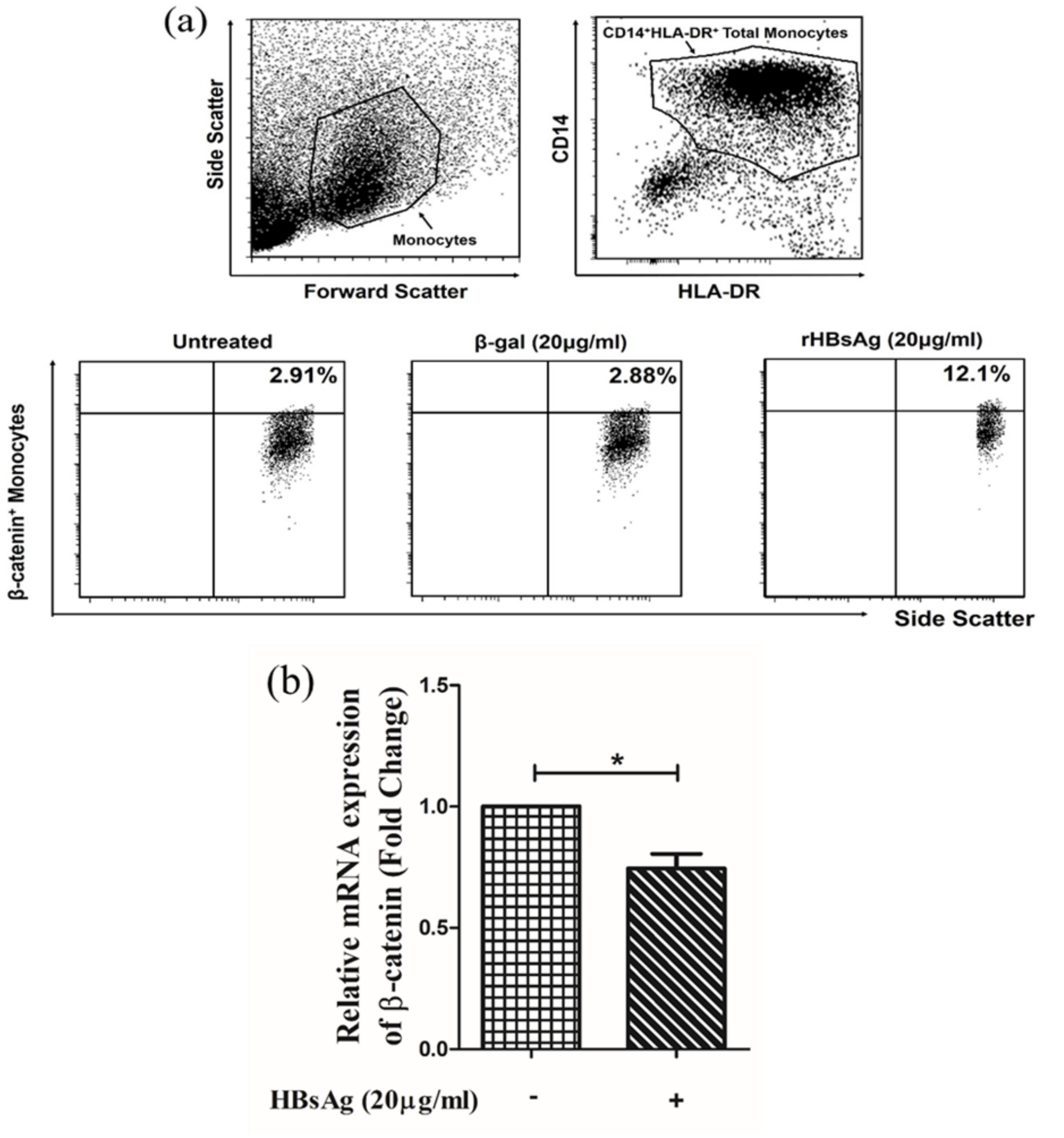

Fig. S6 (a) Representative FACS plots displaying the frequencies of monocytes of healthy controls (HC) expressing β-catenin following treatment of sorted $CD14^+$ monocytes with (a) recombinant Hepatitis B surface antigen (rHBsAg) (20μg/ml) for 48 hours. **(b)** Bar diagram showing relative mRNA expression of β-catenin by real-time PCR in sorted $CD14^+$ monocytes of HC treated with rHBsAg (20ug/ml) for 48 hours. The data was normalized with endogenous 18s ribosomal RNA (rRNA) values. Mean ± SD of three individual set of experiments were given. Statistical significance was analysed using paired t test (**p* <0.05).

Supplementary Fig. S7

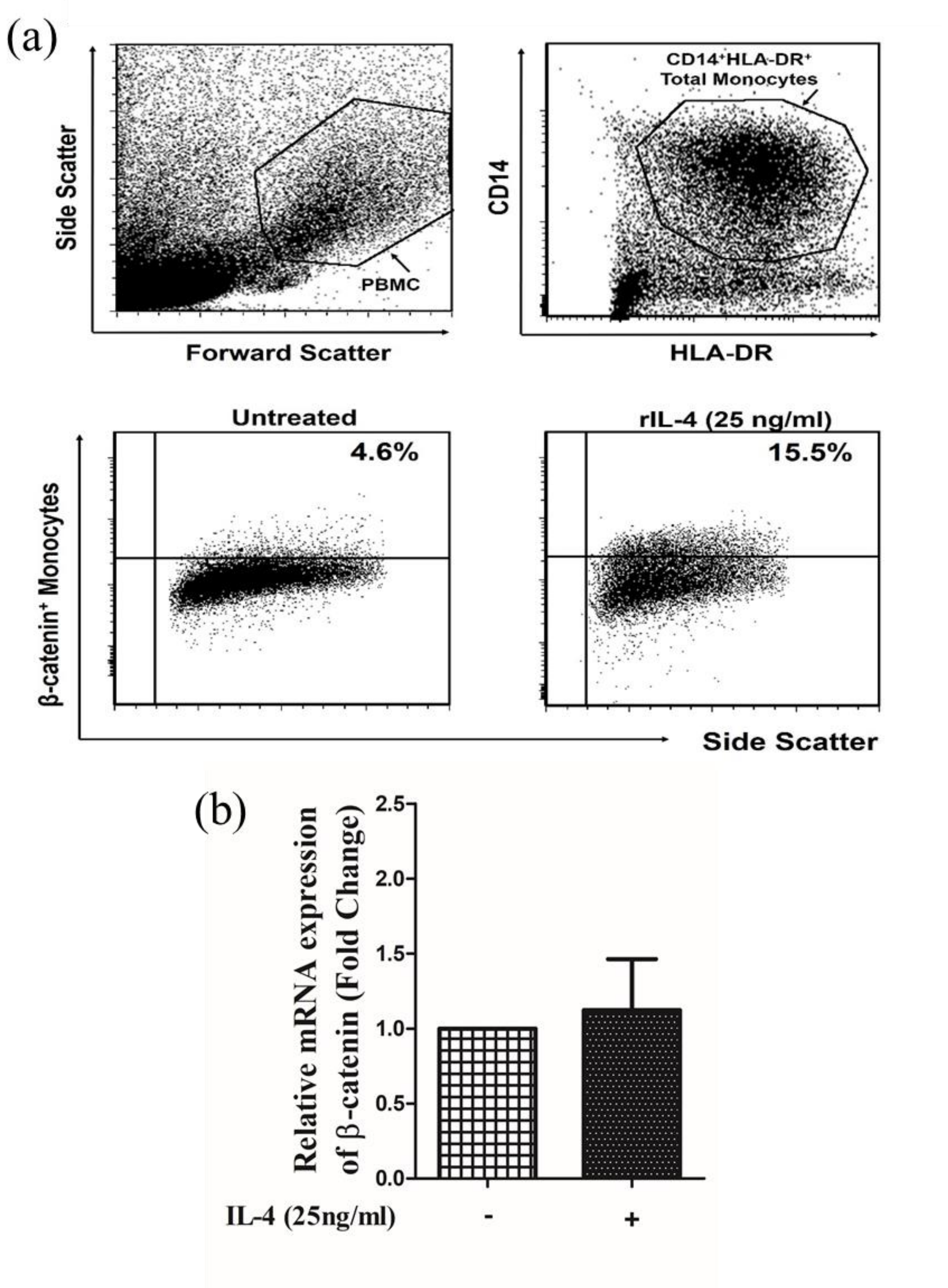

Fig. S7 (a) Representative FACS plots depicting percentages of monocytes of healthy control (HC) following treatment of the PBMC with recombinant IL-4 (rIL-4) (25ng/ml) for 3 days. **(b)** Bar diagram showing relative mRNA expression of β-catenin by real-time PCR in sorted $CD14^+$ monocytes of HC treated with rIL-4 (25ng/ml) for 3 days. The data was normalized with endogenous 18s rRNA values. Mean ± SD of three individual set of experiments were given. Statistical significance was analysed using paired t test.

Supplementary Fig. S8:

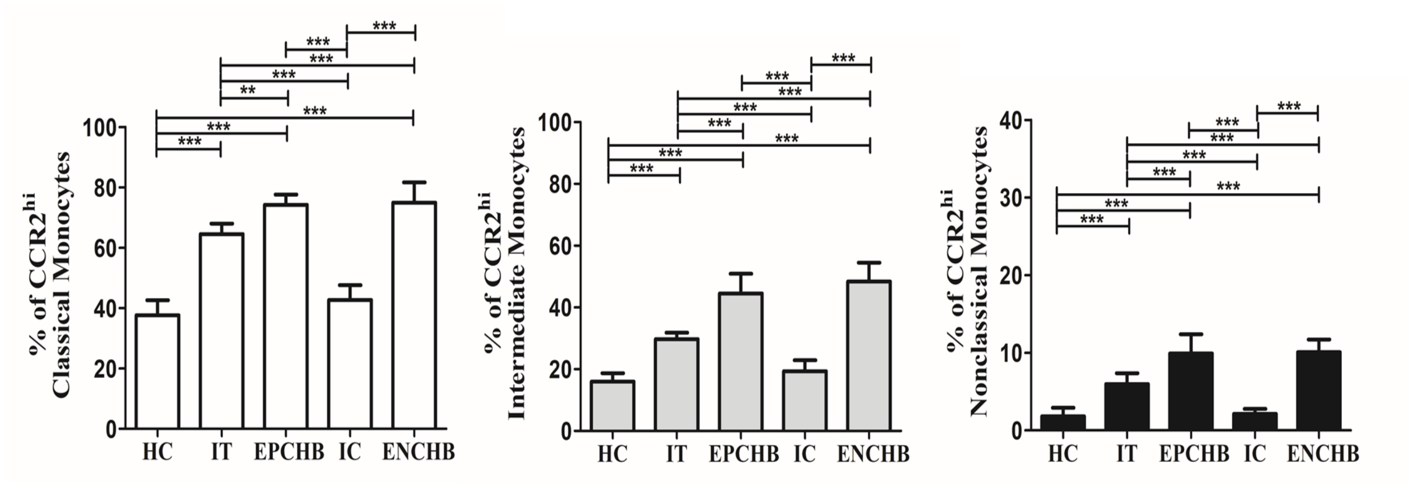

Fig S8: Grouped bar diagrams demonstrating pooled data of percentages of CCR2^{hi} classical, intermediate and non-classical monocytes in healthy controls (HC), Immune-tolerant (IT), HBeAg-positive chronic hepatitis B (EP-CHB), Inactive carriers (IC), and HBeAg-negative chronic hepatitis B (EN-CHB). Statistical significance was assessed by one way ANOVA test followed by Tukey's Multiple Comparison Test. (*$P < 0.05$, **$P < 0.001$ and ***$P < 0.0001$).

Supplementary Fig. S9:

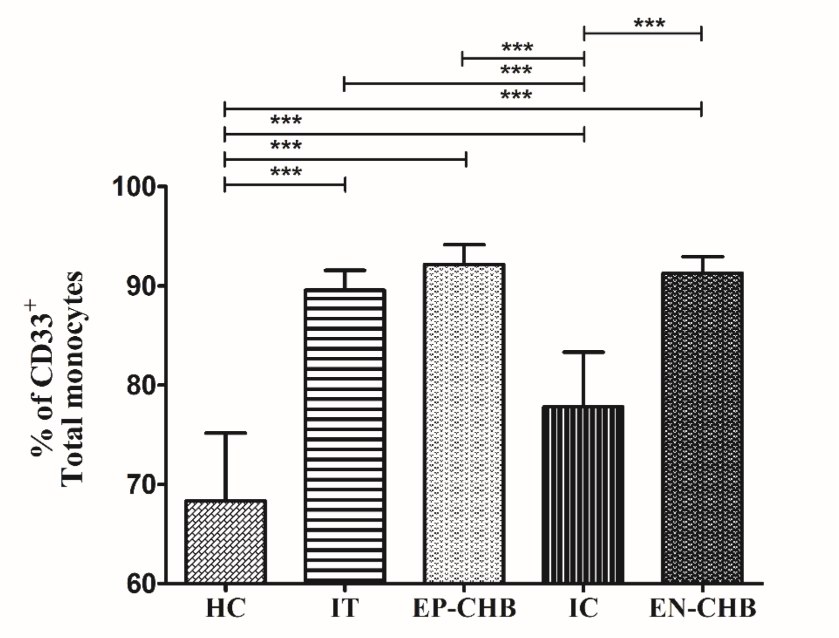

Fig. S9: Grouped bar diagram showing percentages of total monocytes expressing CD33 in healthy controls (HC), Immune-tolerant (IT), HBeAg-positive chronic hepatitis B (EP-CHB), Inactive carriers (IC), and HBeAg-negative chronic hepatitis B (EN-CHB). Statistical significance was assessed by one way ANOVA test followed by Tukey's Multiple Comparison Test. (*P < 0.05, **P < 0.001 and ***P < 0.0001).

Supplementary Fig. S10

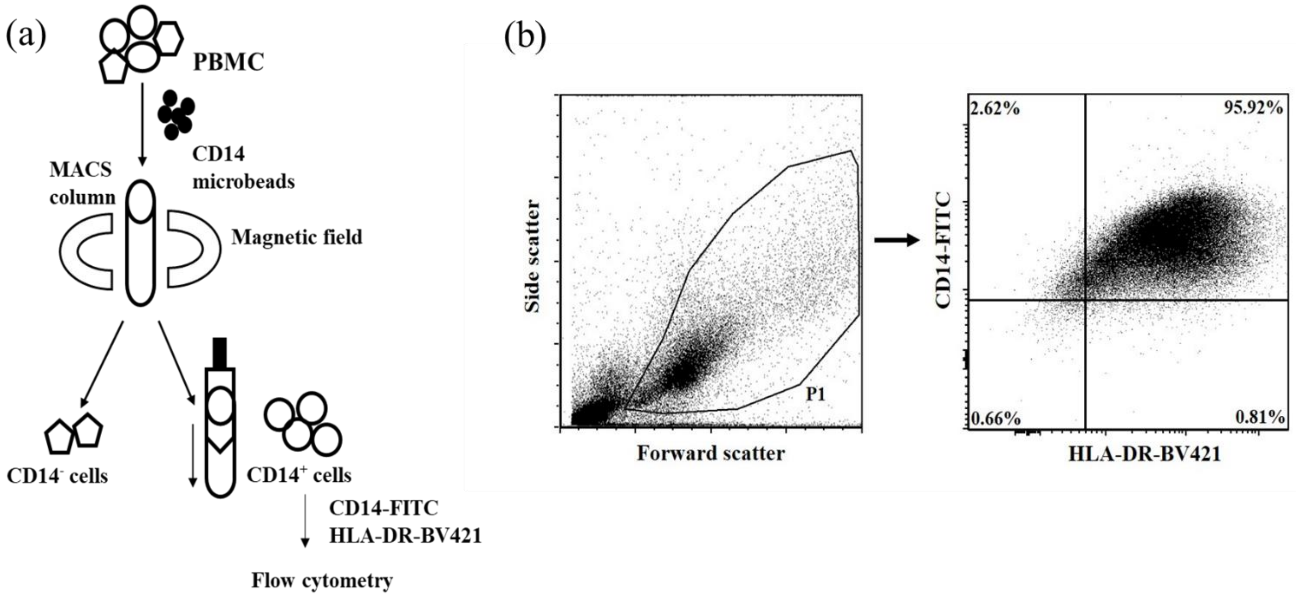

Fig. S10: (a) Flow chart showing MACS sorting and identification of purified monocytes in flow cytometry. PBMCs isolated from the study subjects were incubated with CD14 microbeads followed by washing with PBS containing 0.2% BSA. The cells were then passed through the MS column (Miltenyi Biotec) in a magnetic field. The unbound $CD14^-$ cells were eliminated and the microbead bound $CD14^+$ cells were flushed into the sample collection tube. Next, the cells were washed and stained with fluorochrome conjugated antibodies against CD14 and HLA-DR and acquired on a flow cytometer. **(b)** Representative FACS plot showing purity of sorted $CD14^+HLA\text{-}DR^+$ monocytes. The purity of monocytes was >95%.
